# Supplementary material for: The causal role of circulating inflammatory markers in osteoporosis: a bidirectional Mendelian randomized study
Source: Front Immunol. 2024 Jul 18;15:1412298. doi: 10.3389/fimmu.2024.1412298 (PMC11291241; doi:10.3389/fimmu.2024.1412298)
Supplement: Supplementary file 1 [file DataSheet_1.zip › supplementary material/CIM-OSTEOPOROSIS /GCST90274796.csv_leave_one_out.pdf]

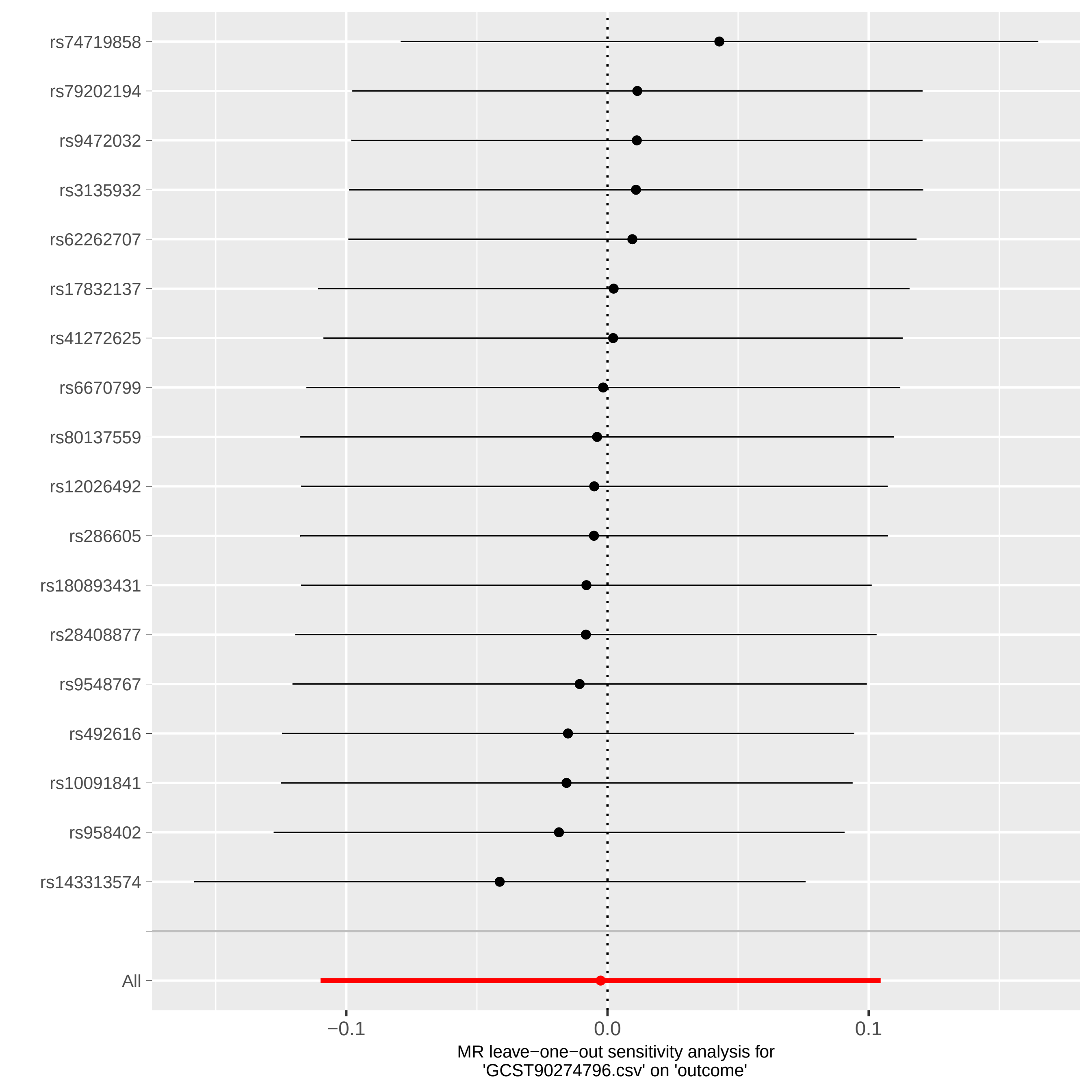
rs74719858
rs79202194
rs9472032
rs3135932
rs62262707
rs17832137
rs41272625
rs6670799
rs80137559
rs12026492
rs286605
rs180893431
rs28408877
rs9548767
rs492616
rs10091841
rs958402
rs143313574
All
−0.1
0.0
0.1
MR leave−one−out sensitivity analysis for
'GCST90274796.csv' on 'outcome'
